# Morphometric and Quantitative Immunohistochemical Analysis of Disease-Related Changes in the Upper (Suburothelial) Lamina Propria of the Human Bladder Dome

**DOI:** 10.1371/journal.pone.0127020

**Published:** 2015-05-14

**Authors:** Thomas Gevaert, Xavier Moles Lopez, Xavier Sagaert, Louis Libbrecht, Tania Roskams, Sandrine Rorive, Christine Decaestecker, Isabelle Salmon, Dirk De Ridder

**Affiliations:** 1 Laboratory of experimental urology, Department of Development and Regeneration, KU Leuven, Leuven, Belgium; 2 Translational Cell and Tissue Research, Department of Imaging and Pathology, KU Leuven, Leuven, Belgium; 3 Department of Pathology, AZ Klina, Brasschaat, Belgium; 4 DIAPath—Center for Microscopy and Molecular Imaging, Université Libre de Bruxelles, Gosselies, Belgium; 5 Department of Pathology, Erasme Hospital, Université Libre de Bruxelles, Brussels, Belgium; 6 Department of Pathology, Ghent University and Ghent University Hospital, Ghent, Belgium; 7 Laboratories of Image, Signal processing and Acoustics, Brussels School of Engineering, Université Libre de Bruxelles, Brussels, Belgium; Temple University School of Medicine, UNITED STATES

## Abstract

The upper (suburothelial) lamina propria (ULP) is a distinct region in the human bladder with dense populations of interstitial cells (IC), fine vascular networks and variable development of muscularis mucosae (MM). It is more and more obvious that the ULP plays an important role in bladder physiology and bladder disease, and in the present study we have quantified changes in the cellular key players of the ULP in bladders from patients with carcinoma in situ (CIS), multiple sclerosis (MS) and bladder pain syndrome (BPS). Tissue samples for the different patient groups were obtained from radical cystectomy-specimens. Standardized immunohistochemistry with a panel of specific cell markers was used to characterise the ULP cellular structures, followed by digitalised morphometry and quantitative staining analysis. Alterations in the ULP area were most pronounced in MS bladders, but also present in BPS and CIS bladders. We observed an increased thickness and increased variability in thickness of the ULP IC area in MS and BPS bladders; a significantly increased development of MM in MS bladders; a changed organization of vascular plexuses in the lamina propria in most pathologic bladders and a changed phenotype of ULP IC: a significantly decreased expression of progesterone receptor in MS bladders and a trend towards decreased expression of alpha-smooth muscle actin in BPS bladders. We show here for the first time the presence of disease-specific changes in organisation and/or phenotype of the different key players of the ULP area in human bladder. The present findings further support the hypothesis that the ULP area is involved and altered in different bladder diseases.

## Introduction

After many years of functional bladder research, it is now obvious that the lamina propria plays an important role in bladder physiology[1]. A particular area of the lamina propria is the upper lamina propria (ULP, also known as suburothelial lamina propria) which houses particular cellular components: fine vascular networks, densely packed interstitial cells and at its depth the presence of muscularis mucosae (MM)[1–3].

It is known since many years that the ULP interstitial cells (IC) (also known as suburothelial IC) have a particular organization and phenotype, but the differences with the underlying deep lamina propria (DLP) have only recently been addressed in several publications[2–4]. Both IC populations are divided by MM, which is variably developed in human bladder. ULP IC are an important research topic in functional bladder research[5, 6], and a constant finding in bladders from rats[5], guinea pigs[6,7] and humans[5, 6]. The functional roles for ULP IC are not well understood yet, some frequent reported findings are myoid differentiation[5, 6], close contacts with afferent nerve endings[8] and a role in cholinergic[7,9] and purinergic[10,11] signalling.

The vascularisation of the LP has been studied in several papers and a suburothelial capillary plexus together with a deeper mucosal vascular plexus have been described[12]. The presence of MM in bladder was first described by Dixon et al.[13], and is defined as the discontinuous smooth muscle layer localized in the middle of the lamina propria. It is only found in human and guinea pig bladder, and its exact function is still unknown.

The involvement of the ULP area in bladder disorders is less explored. In rats with spinal cord injury increased amounts of ULP IC have been reported[10]. In patients with neurogenic detrusor overactivity (NDO) changes in connexin expression have been found[14]. Phenotypes of ULP IC in bladders from patients with NDO and bladder pain syndrome (BPS) were changed towards a fibroblast-like phenotype[15]. In the present study we have focussed on the ULP area in bladders from patients with NDO (in the context of multiple sclerosis (MS)), BPS and carcinoma in situ (CIS)). We focused on the bladder dome, given the increasing evidence for functional differences of the different bladder regions. The ULP area is defined as the area between urothelium and DLP IC, including the ULP IC and the underlying MM. We hypothesised changes in organisation of the major ULP cell types in these diseases since inflammation in the ULP area is a frequent finding in BPS and MS bladders on one hand and given the juxtaposition of this area to the pathologic urothelium in CIS on the other hand.

Immunohistochemistry (IHC) is often used to characterise and study specific cell types and evaluation is mostly performed in a semi-quantitative way, which has the limitation of inter-and intra-observer differences in outcome. The main reasons for this variability are the often partial evaluation of tissue slides (due to evaluation of a fixed amount of high power fields per slide) and the subjective aspect in interpretation of some IHC-parameters like staining intensity. In the present study we have used a standardised and digitalised morphometric technique[16], with the advantages of full evaluation of each tissue sample and solid intensity measurements. We have used this technique to quantify immunohistochemical differences between the ULP key players of the different bladder pathology groups, and we aimed to find disease-specific changes in the ULP area.

## Methods

### 1. Tissue sampling

The study protocol was in accordance with EU guidelines. The study was approved by the ethical committee of the KU Leuven (file number S54423). Informed consent could not be obtained since research was performed on a retrospective tissue database, therefore no waiver was asked to the ethical committee. All patient-related sample-data were fully anonymized in the study analysis. Bladder tissue samples were obtained out of cystectomies from patients with BPS (clinically and histopathologically confirmed, therapy-resistant BPS, disease lasting for more than 5 years, n = 7), MS (clinically confirmed NDO, therapy-resistant NDO, disease lasting for more than 5 years, n = 7) and CIS (persistent CIS or invasive transitional cell carcinoma (TCC), no intravesical/systemic therapies, n = 8). Control tissue came from macroscopically and histologically normal areas out of oncological cystectomies without systemic or intravesical neo-adjuvant treatment (n = 7). These patients did not have any history of clinical bladder dysfunction.

Bladders were opened from the anterior side and full thickness bladder wall biopsies were taken from the bladder dome (in TCC bladders as far as possible from the macroscopically visible tumour). Each biopsy was immediately fixed in formalin 6% and subsequently embedded in paraffin.

### 2. Staining procedure

Before inclusion in the study, paraffin blocks were checked histologically on haematoxylin-eosin staining for the presence of urothelium (at least on parts of the biopsy), since the presence of intact urothelium was a methodological prerequisite for subsequent analysis of the underlying ULP IC and MM. In the CIS group, only ULP areas covered by histologically confirmed CIS were analyzed.

The panel of immunohistochemical markers used was chosen to characterize the ULP area: alpha-smooth muscle actin (alpha-sma) is an established marker for ULP IC[3,4,15]; CD34 is a vascular marker (positive on endothelium[17] and negative on ULP IC[2]); desmin is negative on ULP IC in human bladder and positive on MM[15,18]; progesterone receptor (PR) is positive on ULP IC[19–21] (antibodies are listed in Table 1). Alpha-sma is also expressed on MM, but areas of MM were not included in the analysis of apha-sma+ ULP IC (see below and S1 Fig). The specificity of all antibody clones used was approved by NordiQC (independent scientific organisation for immunohistochemical quality control (www.nordiqc.com)).

**Table 1:** Table listing the details of the antibodies used in the present study.

Antibody	Manufacturer	Host	Clone	Antibody registry N°	Titer
Alpha-sma	Dako, Glostrup, Denmark	Mouse	1A4	AB_2341211	RTU
CD34	Dako, Glostrup, Denmark	Mouse	Qbend 10	AB_2063006	RTU
Progesterone receptor	Dako, Glostrup, Denmark	Mouse	PGR636	AB_2252608	RTU
Desmin	Dako, Glostrup, Denmark	Mouse	D33	AB_2335684	RTU

RTU equals ready to use.

Paraffin blocks were cut in serial slides of 5μm to have all immunohistochemical stainings on similar tissue areas. All immunohistochemical stainings were done on the Dako autostainer (Dako, Glostrup, Denmark). For each antibody, slides were stained as much as possible in one batch; when additional batches were needed control slides of the first batch were always included to normalize the staining parameters (see below). The staining procedure consisted of the following algorithm: heat induced antigen retrieval with citrate buffer at pH 6.1, incubation with Envision FLEX Peroxidase-Blocking Reagent for 5 minutes, incubation with the primary antibody for 30 minutes, incubation with the secondary antibody coupled with the activation reagent EnVision FLEX HRP for 20 minutes and visualization with EnVision FLEX DAB+ chromogen (Incubation time 10 minutes). Between all steps slides were washed with Dako rinse buffer. Nuclear counterstaining was done with haematoxylin. Images were viewed using a Leica DM LB microscope equipped with a DC300FX camera (Leica Microsystems, Belgium).

### 3. Morphometry and quantitative staining analysis

Within 2 weeks after staining slides were digitalised using the NanoZoomer 2.0-HT slide scanner (Hamamatsu, Hamamatsu City, Japan), to avoid bleaching of the stains. Annotation was performed using the NDP. View viewer software (Hamamatsu) following stringent parameters (see S1 Fig). Only ULP areas covered by urothelium were annotated. The ULP IC area was defined as the area of sma+/CD34- ULP IC between urothelium and muscularis mucosae (MM) (based on the current knowledge of ULP IC phenotypes)[2, 3, 4]. Only areas with ULP IC oriented horizontally to the urothelium were annotated (to avoid tangential effects). Similar ULP IC areas were annotated for alpha-sma, CD34 and PR in serial slides. In a second phase MM was annotated, only in areas with intact urothelium, and both areas with and without MM were annotated. MM was considered as all desmin+ smooth muscle fibers in the LP, localized directly underneath the ULP IC (see S1 Fig).

The manual ULP IC and MM annotations were then extracted to bitmap images using homemade software. We used 2 measures to calculate the thickness of the ULP IC area: mean area (MA) and mean thickness (MTH) (for detailed information see S2 Fig and S1 File). Finally, the standard deviation of the ULP IC area thickness was calculated (STD_TH) (for detailed information see S1 File).

A quantitative staining analysis was then performed using the Visiomorph software package (Visiopharm, Hoersholm, Denmark). For each immunohistochemical marker we computed the labeling index (LI), the mean density (MD) and the Quick score (QS) in the regions of interest (annotations). As detailed previously[16], the LI is the percentage of the immunostained tissue area, whereas MD and QS are two different measures of the staining intensity (for both features low vs. high values indicate weak vs. strong staining). While MD is the mean staining intensity computed on the positive tissue area only, QS takes the extent of the positive tissue area into account by averaging the staining intensity on all the tissue area under analysis (where the negative tissue pixels were considered as having a null staining intensity). For PR, which exhibited a nuclear staining, LI, MD and QS were computed on the nuclear area only[16]. We observed that the lymphocytes that infiltrated ULP IC areas (in various extents depending of the disease) could strongly impact the PR-negative nuclear areas taken into account in the PR-LI and QS measurements. This is the reason why for the PR measurements we excluded the lymphocyte infiltrates from the regions of interest.

### 4. Statistical analysis

Independent groups of quantitative data were compared using the non-parametric Kruskal-Wallis test. In case of significance (p < 0.05) this test was completed by post-hoc tests to compare pairs of groups. The p-values indicated in the results are those provided by these pair comparisons.

## Results

**Fig 1:**
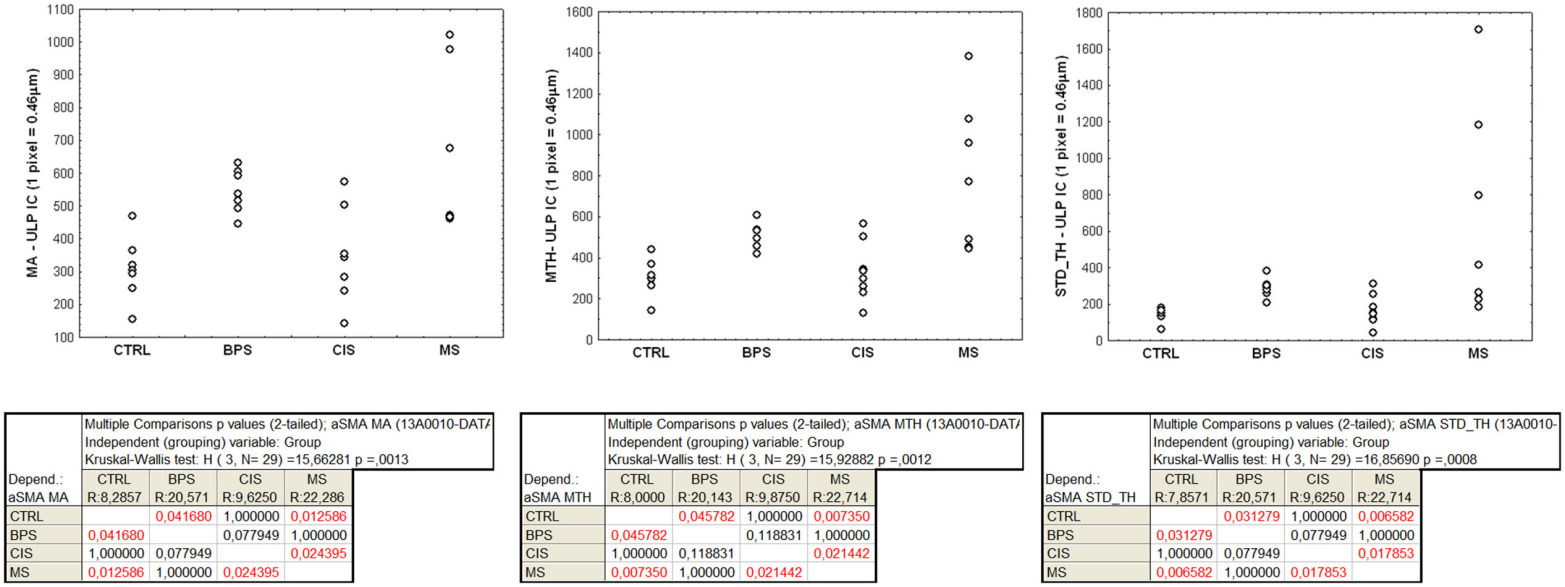
Data distributions observed across the 4 pathological groups for the thickness-related measures (MA, MTH and STD_TH), which are expressed in pixels (1 pixel = 0.46μm). Each dot shows the value associated with one case. The results of the Kruskal-Wallis test and the post-doc pair comparison test are shown below.

**Fig 2:**
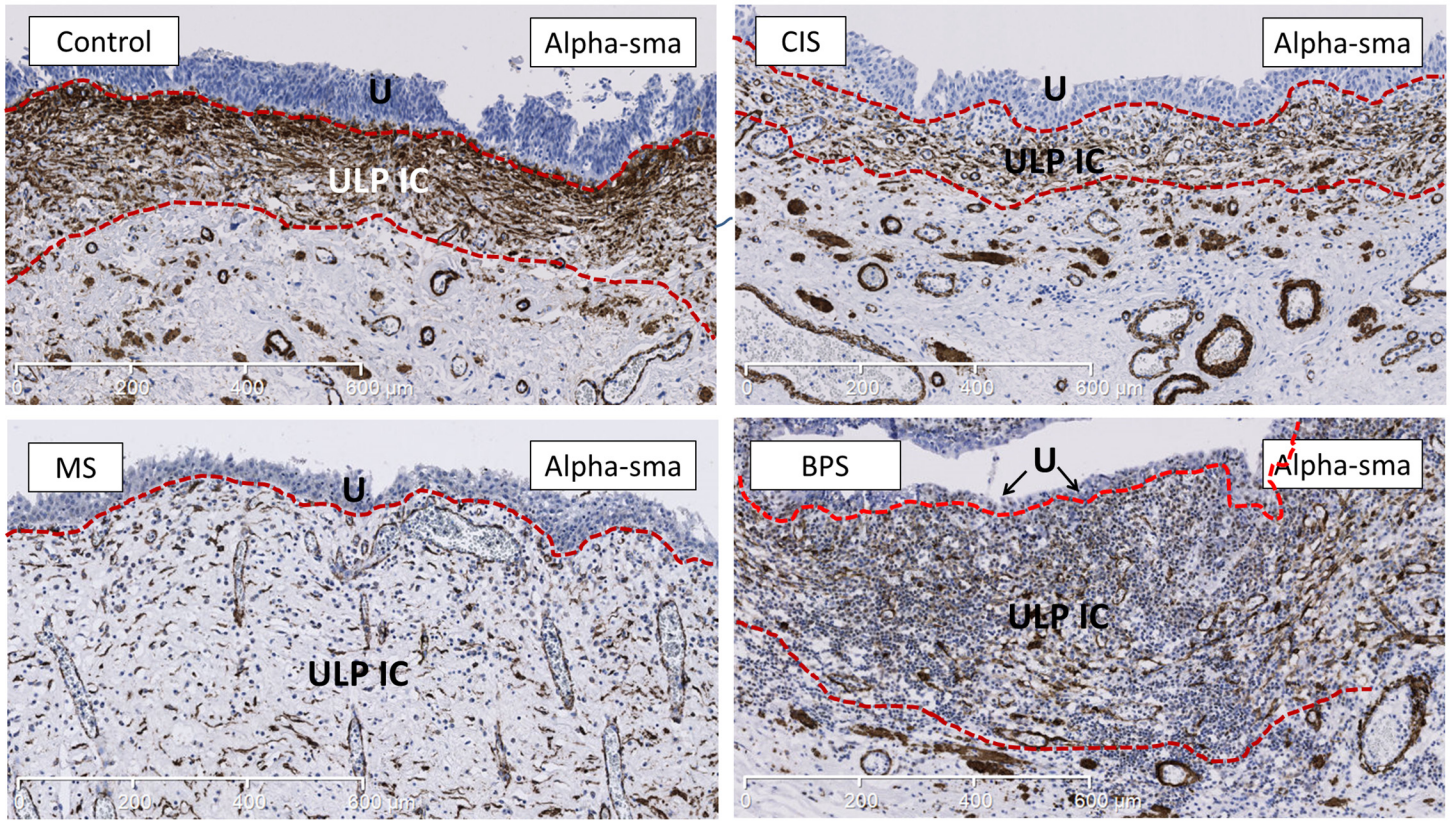
Representative images of staining for alpha-sma (marker for ULP IC) showing the increased thickness of the alpha-sma+ ULP IC area in MS and BPS bladders. The ULP IC area lies between the 2 dotted red lines. The alpha-sma+ cell-types under the ULP IC area are MM-fibres and perivascular smooth muscle cells (which were excluded from analysis); IC in the DLP are negative for alpha-sma. In BPS bladder heavy inflammatory infiltrate is laying in between the IC, while in MS bladder this infiltrate is less pronounced. U is urothelium, ULP IC equals upper lamina propria interstitial cell. Scale bars indicate 600μm.

The two thickness measures (MA and MTH) extracted from the manual annotations confirmed that the ULP IC area (= area of alpha-sma+/CD34- ULP IC) was significantly thicker in MS bladders compared to controls (p = 0.01) and CIS (p = 0.02), and in BPS bladders compared to controls (p = 0.04). There was a significantly increased heterogeneity in thickness of the ULP IC area (calculated by means of the STD_TH measurement in the sma-annotations) in MS bladders compared to controls (p = 0.007) or CIS (p = 0.02) and in BPS bladders compared to controls (p = 0.03) (Figs 1 and 2).

**Fig 3:**
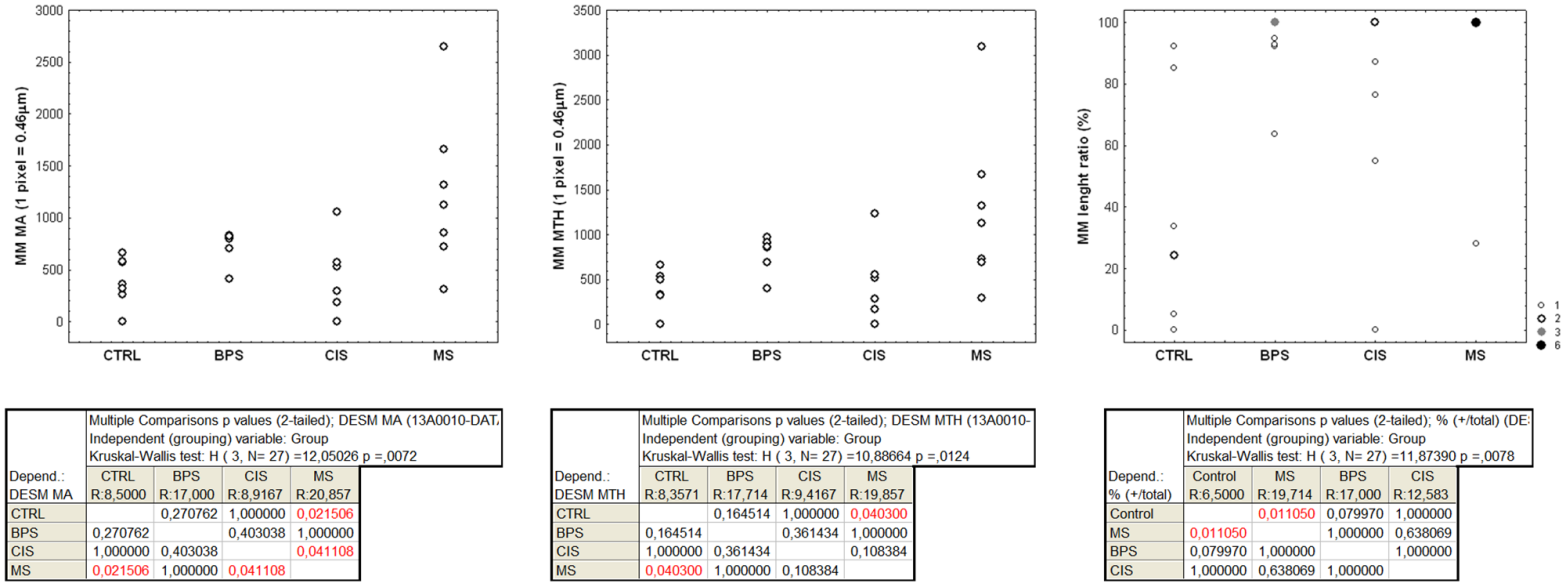
Data distributions observed across the 4 pathological groups for the MM thickness-related measures (MA and MTH), which are expressed in pixels (1 pixel = 0.46μm), and the length ratio (length of MM / total length of ULP IC area, expressed in %). For the MA and MTH data each dot shows the value associated with one case, whereas frequency-related sizes are used for the dots showing the length ratio data. The results of the Kruskal-Wallis test and the post-doc pair comparison test are shown below.

**Fig 4:**
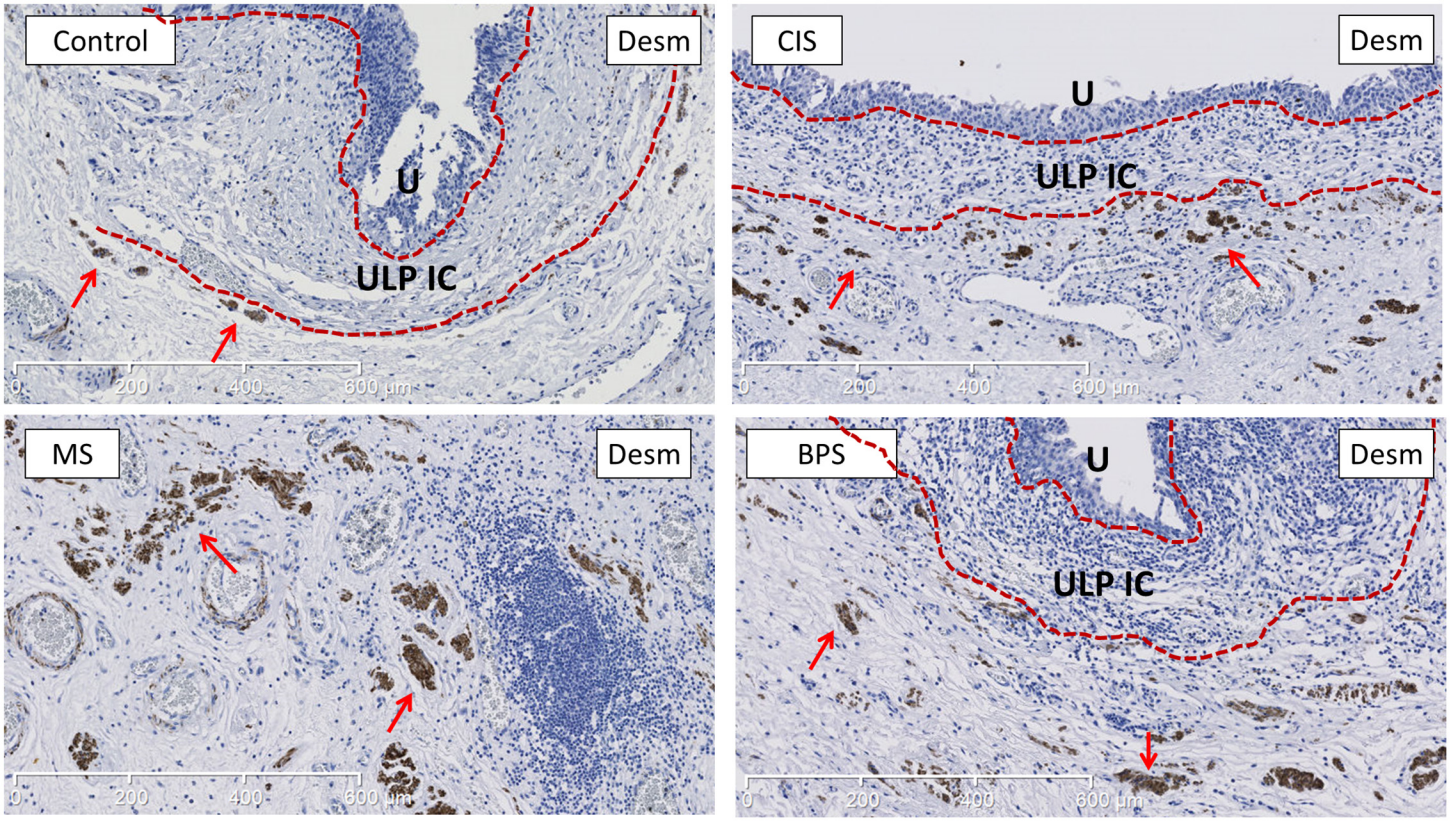
Representative images of desmin-staining (marker for MM) showing the increased development of desmin+ MM in MS bladders compared to controls. MM is more developed in MS bladders, both vertically and horizontally. MM (indicated by red arrows) lies just below the ULP IC area (between the 2 dotted red lines). In BPS and CIS bladders there was a trend towards increased MM development, but no significant difference was found (see Fig 3). Scale bars indicate 600μm. U is urothelium, ULP IC equals upper lamina propria interstitial cells.

The presence of MM was characterized by thickness and length ratio (length of MM/total length of ULP IC area). The MM thickness measurements (both MA and MTH) were significantly increased in MS compared to control bladders (p < 0.05). Similarly, the MM length ratio was significantly increased in MS compared to control bladders (p = 0.01); a similar upward trend was observed in BPS bladders (p = 0.08) (Figs 3 and 4).

Concerning the staining analysis, because the MD feature (i.e. mean intensity in the positive area) for alpha-sma and PR did not show significant variations across groups, the LI and the QS features are strongly correlated and show similar data distributions. Therefore, only the LI (% of stained area) results are described and illustrated.

**Fig 5:**
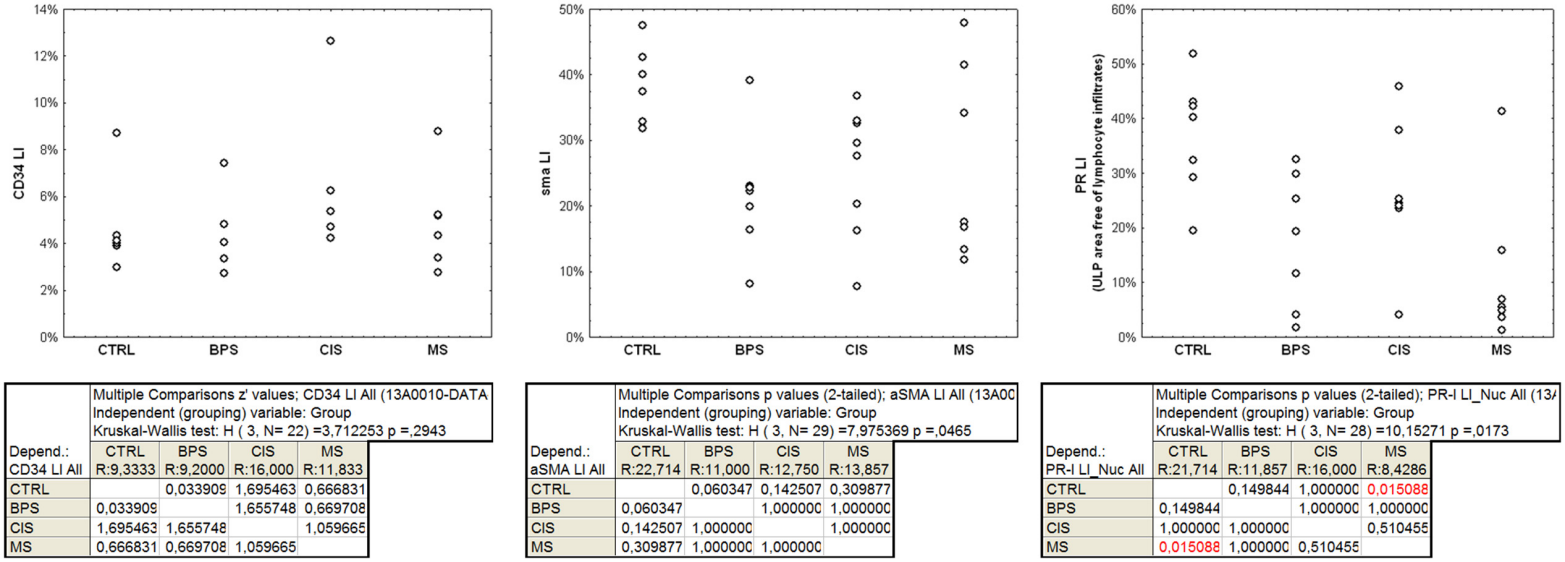
Quantitative evaluation of the percentage of tissue area exhibiting CD34 or sma immunopositivity and of nuclear area exhibiting PR immunopositivity (LI, labeling index) in the ULP area. Each dot shows the value associated with one case. The results of the Kruskal-Wallis test and the post-doc pair comparison test are shown below.

**Fig 6:**
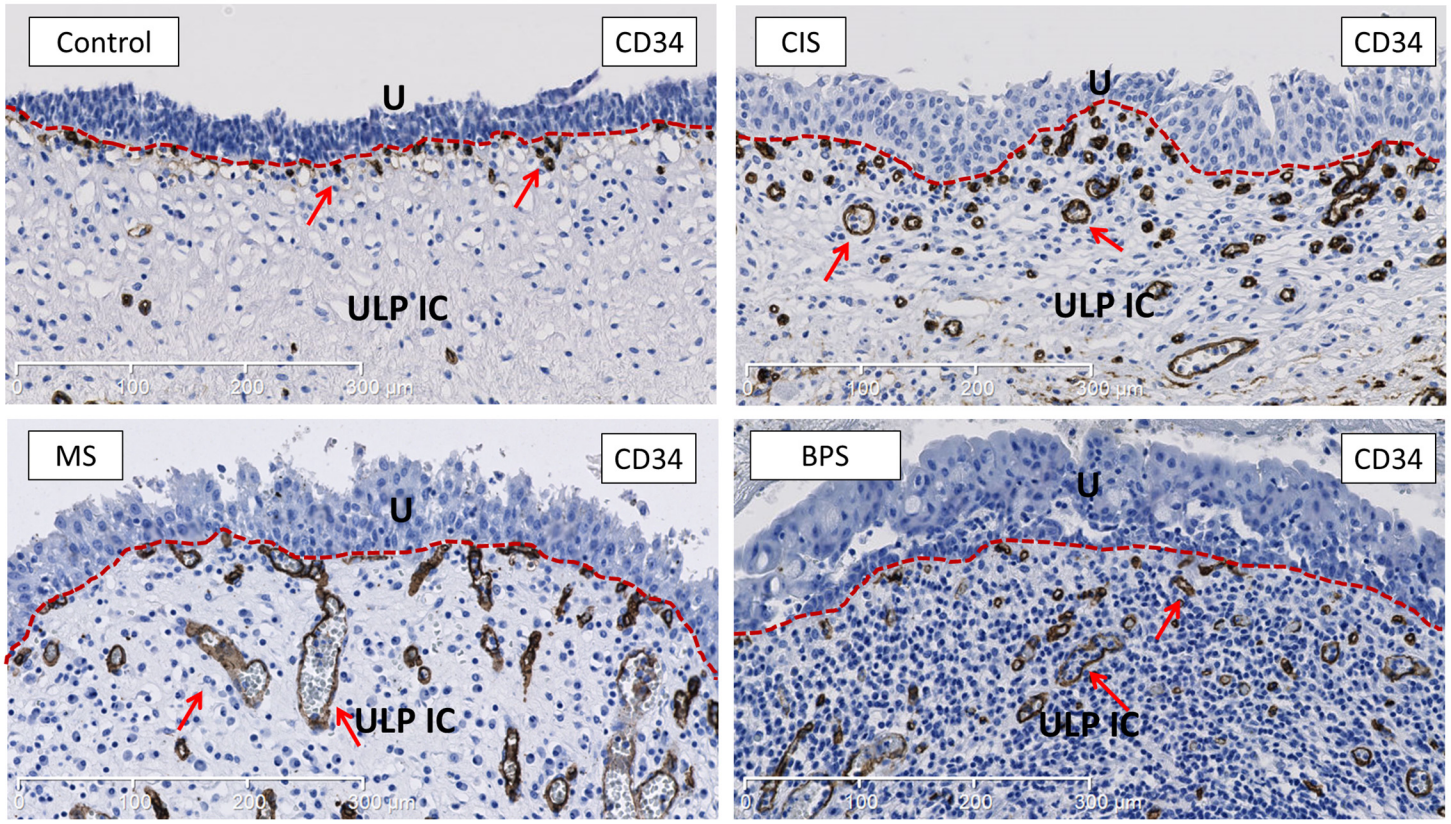
Representative images of CD34-staining (endothelial marker) showing the vascularisation of the bladder ULP area in the different studied groups. No significant difference was found in total vascularisation in any of the studied groups (see Fig 5). As shown in the images the vascular topography was changed in all bladder disease groups, with blood vessels being organised more haphazardly in the ULP, although this observation could not be quantified. Arrows indicate CD34+ blood vessels and capillaries. U is urothelium, ULP IC equals upper lamina propria interstitial cells (red dotted line separates urothelium form the ULP IC area). Scale bars indicate 300μm.

The amount of CD34+ vessels in the ULP area was not significantly changed in BPS, MS and CIS bladders compared to controls (LI, calculated by the percentage of CD34-positive tissue area in the ULP area) (Figs 5 and 6). In most pathological bladder samples the organization in suburothelial and mucosal plexuses was disturbed, but this observation was difficult to quantify. We further observed a downward trend in sma protein expression (LI) in ULP IC in BPS bladders compared to controls (p = 0.06). Due to data heterogeneity, these latter results should be validated on a larger series (Figs 2 and 5).

**Fig 7:**
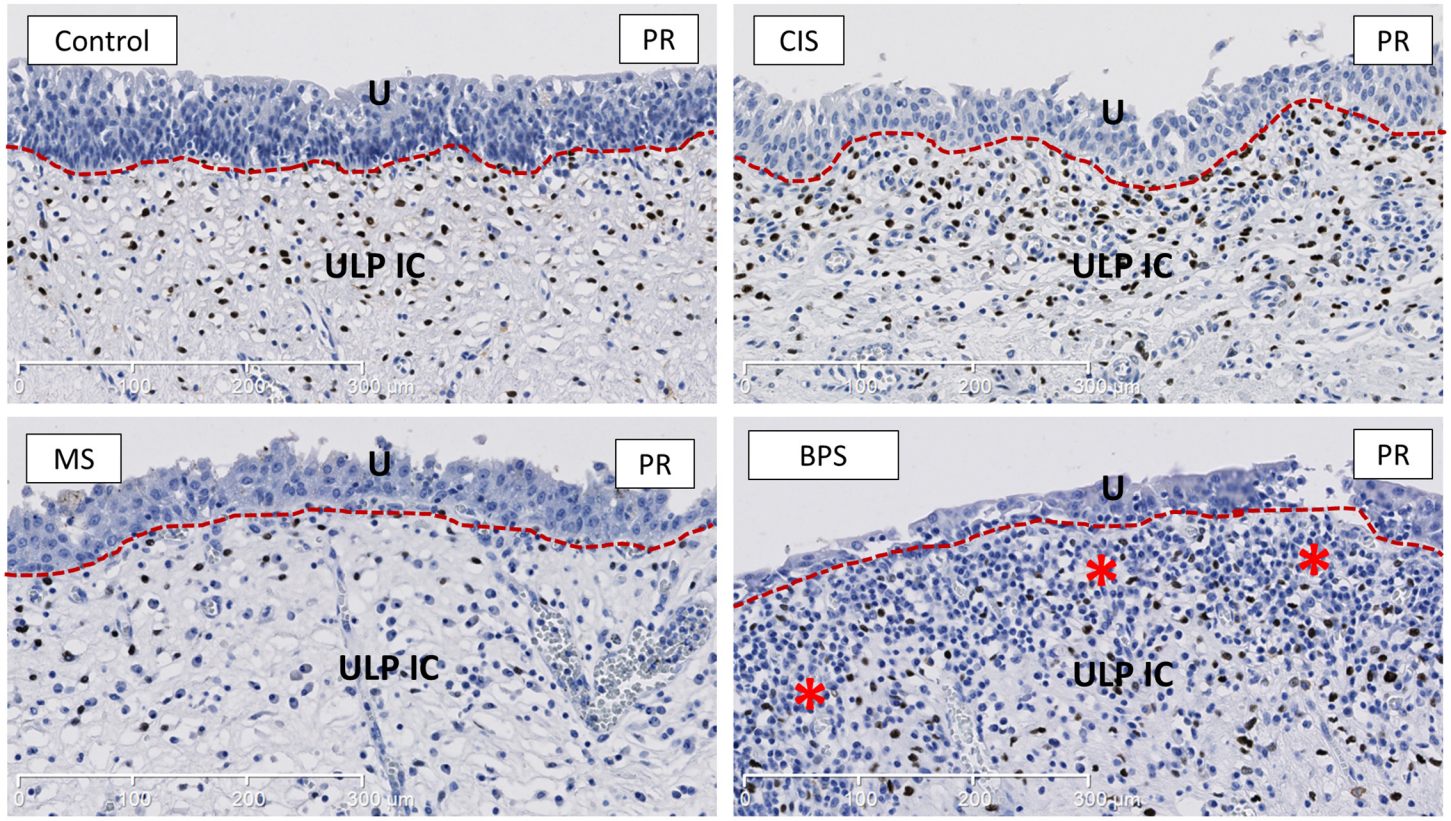
Representative images of PR-staining, showing the decreased amount of PR+ ULP IC in MS bladders. PR shows a nuclear expression and is widely expressed in ULP IC. Asterisks indicate heavy inflammatory infiltrate in the ULP area in BPS. U is urothelium, ULP IC equals upper lamina propria interstitial cells (red dotted line separates urothelium form the ULP IC area). Scale bars indicate 300μm.

The amount of PR+ ULP IC (Li, calculated by the percentage of PR staining/total nuclear area in the ULP area free of lymphocyte infiltrates) was significantly decreased in MS compared to controls (p = 0.02) (Figs 5 and 7).

## Discussion

In the present study we have investigated the ULP area in different human bladder diseases with a standardized quantitative morphometric approach. This methodology is complex and time-consuming, and it could be questioned why we did not choose semi-quantitative evaluation of IHC. The latter approach has several limitations compared to the present methodology: intra- and inter-observer differences in interpretation of areas and intensities of staining (the current interpretation is standardized and automatized) and the limited amount of tissue area that is evaluated (areas of interest are now fully evaluated, which generates reproducible data). Another criticism on the present study could be the limited amount of samples/group (n = 7–8/group). We have chosen to work with full thickness samples dissected from cystectomies, which significantly limits the available patient pool (due to the limited amount of cystectomies in these patient populations). The experimental rationale was that with cystectomies we were able to follow a standardized dissection protocol which provided us with large tissue samples all taken from similar bladder areas. The limited group size is partially overcome by the analysis of the complete area of interest/sample, which resulted in solid and reproducible data/sample (in contrast with at random selected areas in a semi-quantitative analysis).

We have observed several differences in the ULP IC area: increased thickness and increased variability in thickness of the ULP IC area in MS and BPS compared to controls and CIS, most pronounced in MS bladders. In MS bladders changes in organization of IC are likely to be responsible for these observations: previous work has shown a change in phenotype of the ULP IC (more fibroblast-features, less contractile filaments) with an associated increase in intercellular matrix between ULP IC[15], which might be the primary etiologic factor for the increased width of the ULP area. In BPS bladders the high amount of inflammatory cells in the ULP IC area is likely to be the major determining factor. In CIS one would expect an increased width of the ULP IC area due to development of reactive tumor stroma, but this was not found. The increased variability in ULP IC area thickness in MS and BPS bladders suggests multifocality of disease-related changes, resulting in variable severity of pathological involvement.

We have tried to quantify changes in the phenotypes of ULP IC by means of 2 ULP IC markers. Alpha-sma is present on ULP IC and not on DLP IC [2, 3, 19]. PR is widely expressed on ULP IC[15,20,21] (not on any other ULP cell type), but can also been found on some DLP IC (which was excluded from the current analysis). Alpha-sma showed a trend towards decreased expression in BPS bladders and for some cases of MS bladders (no significant difference was found, but larger sample series might resolve this problem); PR-expression was significantly decreased in MS bladders. The trend towards decreased alpha-sma expression is in line with previous reports in MS and BPS bladders where a shift from myoid to fibroblast-phenotype for ULP IC has been observed[15]. Such decrease in myoid phenotype might have repercussions on the role of ULP IC in modulating bladder contractile function. PR-expression on ULP IC is a relatively recent finding[15,20,21], and its role in bladder function remains to be elucidated. It is known that bladder function depends on estrogen and progesterone levels: postmenopausal woman have a decreased bladder function which ameliorates after estrogen supplementation[22]. Progesterone is thought to play an active role in steroid hormone dependent regulation of bladder function via direct or indirect (inhibition of estrogen-effects) pathways[20]. A decreased expression of PR in MS bladders is likely to alter progesterone-driven bladder regulation, which might then contribute to the NDO observed in these patients.

We have assessed the degree of vascularization in the ULP IC area by means of quantification of expression of the endothelial marker CD34[17] (which was not expressed by any other cell type in the ULP IC area). No significant increase was found in any of the pathological groups. For BPS bladder these findings are in line with previous reports[23], for MS and CIS no previous studies have been found. In inflammatory[24] and oncological[25] conditions angiogenesis is a widely described phenomenon. The present findings are not in contradiction with angiogenesis, but we could not find an increase in absolute amount of blood vessels in the ULP IC area. Although not quantifiable, the organization in a superficial and deep vascular plexus in the lamina propria was altered in most pathologic bladders. This might indicate that in BPS, MS and CIS there is angiogenesis: not an absolute increase in vascularization (as observed in the present study and by others[23]), but a changed topographical distribution of capillaries and blood vessels in the ULP IC area.

To evaluate the MM we used desmin, which is known to be expressed on MM, but not on any other cell type in the ULP or DLP in the human bladder [18] (this contrasts with recent reports of desmin-expression on DLP IC in mouse bladder[4], a discrepancy possibly related to inter-species differences). We did observe a significantly increased development of MM in bladders from patients with MS, both in thickness and length (in BPS and CIS a slight trend towards increased MM development was observed). Proposed roles for MM are synthesis and secretion of extracellular matrix constituents and generation of contractions, possibly contributing to the non-voiding contractions[1]. In MS bladders this increased MM is probably a secondary phenomenon following neurological damage, but it might then contribute to the NDO-pattern by matrix production or generation of additional contractions[1].

In conclusion this is the first study to evaluate the ULP (suburothelial) area in different bladder pathologies with a quantitative methodology. Changes have been found in organisation and phenotypes of ULP IC, degree of vascularisation and development of MM in MS and BPS bladders, while in CIS no significant alterations in the ULP area were observed. The functional roles for the different components of the ULP area remain largely unclear, but the distinct cellular organisation and particular ULP IC phenotypes suggest a different role for the ULP compared to the DLP. The present findings suggest an active role for the ULP area in inflammatory/functional bladders diseases like BPS and MS, which seems to be less pronounced in CIS. Future research should further focus on the complex interplays between the different key players of the ULP area to get better insights in the role of the ULP in bladder disease.

## Supporting Information

S1 Fig Illustrative figure for the annotation methodology. Illustrative figure that shows the annotation methodology for SMA+/CD34-/PR+ ULP IC, CD34+ blood vessels and desmin+ MM in the ULP area (all illustrations come from the same sample area). ULP IC areas are encircled by black diagrams. For SMA, PR and CD34 the same ULP IC areas were annotated on images of serial tissue slides, while for desmin the underlying presence or absence of MM was annotated with free hand diagrams and rulers respectively. The area on the present tissue slide shows absence of MM, resulting in annotation with rulers only. Black and red arrows indicate the differences between ULP IC and DLP IC respectively. All annotations were extracted in a second phase for morphometry and quantitative staining analysis. U is urothelium. Scale bars indicate 600μm.
(TIF)

S2 Fig Method for extracting thickness measures. (A) ULP IC annotation extracted as a bitmap image. (B) Binary mask (*m*) identifying ULP IC area (in white on a black background). (C) Distance transform applied on image *m* to produce image *d* and its skeleton as the ridges of the distance function (overlaid in red).
(TIFF)

S1 File Detailed technical methodology for calculation of the thickness of the ULP IC area. (DOCX)

## References

[1] Andersson K, McCloskey KD. Lamina propria: The functional center of the bladder? Neurourology and urodynamics. 2013;33(1):9–16. 10.1002/nau.22465 23847015

[2] Gevaert T, Vanstreels E, Daelemans D, Franken J, van der Aa F, Roskams T, et al Identification of different phenotypes for the interstitial cells in the upper and deep lamina propria of the dome of the human bladder. The Journal of urology. 2014;192(5):1555–63. 10.1016/j.juro.2014.05.096 24893312

[3] Vannucchi M, Traini C, Guasti D, del Popolo G, Faussone-Pellegrini M. Telocytes subtypes in human urinary bladder. Journal of cellular and molecular medicine. 2014;18(10):2000–8. 10.1111/jcmm.12375 25139461 PMC4244015

[4] Yu W, Zeidel ML, Hill WG. Cellular expression profile for interstitial cells of cajal in bladder—a cell often misidentified as myocyte or myofibroblast. PloS one. 2012;7(11):e48897 10.1371/journal.pone.0048897 23145014 PMC3492220

[5] Gevaert T, Hutchings G, Everaerts W, Prenen H, Roskams T, Nilius B, et al Administration of Imatinib Mesylate in Rats Impairs the Neonatal Development of Intramuscular Interstitial Cells in Bladder and Results in Altered Contractile Properties. Neurourology and urodynamics. 2014;33(4):461–8. 10.1002/nau.22415 23616342

[6] Monaghan KP, Johnston L, McCloskey KD. Identification of PDGFRα positive populations of interstitial cells in human and guinea pig bladders. The Journal of urology. 2012;188(2):639–47. 10.1016/j.juro.2012.03.117 22704452

[7] Grol S, Essers PB, van Koeveringe GA, Martinez-Martinez P, de Vente J, Gillespie JI. M(3) muscarinic receptor expression on suburothelial interstitial cells. BJU international. 2009;104(3):398–405. 10.1111/j.1464-410X.2009.08423.x 19338557

[8] Wiseman OJ, Fowler CJ, Landon DN. The role of the human bladder lamina propria myofibroblast. BJU international. 2003;91(1):89–93. 12614258 10.1046/j.1464-410x.2003.03802.x

[9] Mukerji G, Yiangou Y, Grogono J, Underwood J, Agarwal SK, Khullar V, et al Localization of M2 and M3 muscarinic receptors in human bladder disorders and their clinical correlations. The Journal of urology. 2006;176(1):367–73. 16753445 10.1016/S0022-5347(06)00563-5

[10] Fry CH, Young JS, Jabr RI, McCarthy C, Ikeda Y, Kanai AJ. Modulation of spontaneous activity in the overactive bladder: the role of P2Y agonists. American journal of physiology. Renal physiology. 2012;302(11):F1447–54. 10.1152/ajprenal.00436.2011 22357922 PMC3378171

[11] Sui G, Wu C, Fry CH. Characterization of the purinergic receptor subtype on guinea-pig suburothelial myofibroblasts. BJU international. 2006;97(6):1327–31. 16686733 10.1111/j.1464-410X.2006.06200.x

[12] Miodoński AJ, Litwin JA. Microvascular architecture of the human urinary bladder wall: a corrosion casting study. The Anatomical record. 1999;254(3):375–81. 10096669 10.1002/(SICI)1097-0185(19990301)254:3<375::AID-AR8>3.0.CO;2-R

[13] Dixon JS, Gosling JA. Histology and fine structure of the muscularis mucosae of the human urinary bladder. Journal of anatomy. 1983;136(Pt 2):265–71. 6682849 PMC1170972

[14] Roosen A, Datta SN, Chowdhury RA, Patel PM, Kalsi V, Elneil S, et al Suburothelial myofibroblasts in the human overactive bladder and the effect of botulinum neurotoxin type A treatment. European urology. 2009;55(6):1440–8. 10.1016/j.eururo.2008.11.009 19054608

[15] Gevaert T, de Vos R, Everaerts W, Libbrecht L, van der Aa F, van den Oord J, et al Characterization of upper lamina propria interstitial cells in bladders from patients with neurogenic detrusor overactivity and bladder pain syndrome. Journal of cellular and molecular medicine. 2011;15(12):2586–93. 10.1111/j.1582-4934.2011.01262.x 21251216 PMC4373427

[16] Decaestecker C, Lopez XM, D'Haene N, Roland I, Guendouz S, Duponchelle C, et al Requirements for the valid quantification of immunostains on tissue microarray materials using image analysis. Proteomics. 2009;9(19):4478–94. 10.1002/pmic.200800936 19670370

[17] Pusztaszeri MP, Seelentag W, Bosman FT. Immunohistochemical expression of endothelial markers CD31, CD34, von Willebrand factor, and Fli-1 in normal human tissues. The journal of histochemistry and cytochemistry: official journal of the Histochemistry Society. 2006;54(4):385–95. 16234507 10.1369/jhc.4A6514.2005

[18] Council L, Hameed O. Differential expression of immunohistochemical markers in bladder smooth muscle and myofibroblasts, and the potential utility of desmin, smoothelin, and vimentin in staging of bladder carcinoma. Modern pathology: an official journal of the United States and Canadian Academy of Pathology, Inc. 2009;22(5):639–50. 10.1038/modpathol.2009.9 19252475

[19] Gevaert T, de Vos R, van der Aa F, Joniau S, van den Oord J, Roskams T, et al Identification of telocytes in the upper lamina propria of the human urinary tract. Journal of cellular and molecular medicine. 2012;16(9):2085–93. 10.1111/j.1582-4934.2011.01504.x 22151349 PMC3822978

[20] Tincello DG, Taylor AH, Spurling SM, Bell SC. Receptor isoforms that mediate estrogen and progestagen action in the female lower urinary tract. The Journal of urology. 2009;181(3):1474–82. 10.1016/j.juro.2008.10.104 19157432

[21] Blakeman PJ, Hilton P, Bulmer JN. Oestrogen and progesterone receptor expression in the female lower urinary tract, with reference to oestrogen status. BJU international. 2000;86(1):32–8. 10886079 10.1046/j.1464-410x.2000.00724.x

[22] Cardozo L, Lose G, McClish D, Versi E. A systematic review of the effects of estrogens for symptoms suggestive of overactive bladder. Acta obstetricia et gynecologica Scandinavica. 2004;83(10):892–7. 15453881 10.1111/j.0001-6349.2004.00581.x

[23] Kiuchi H, Tsujimura A, Takao T, Yamamoto K, Nakayama J, Miyagawa Y, et al Increased vascular endothelial growth factor expression in patients with bladder pain syndrome/interstitial cystitis: its association with pain severity and glomerulations. BJU international. 2009;104(6):826–31; discussion 831. 10.1111/j.1464-410X.2009.08467.x 19298410

[24] Chidlow JH Jr, Shukla D, Grisham MB, Kevil CG. Pathogenic angiogenesis in IBD and experimental colitis: new ideas and therapeutic avenues. American journal of physiology. Gastrointestinal and liver physiology. 2007;293(1):G5–G18. 17463183 10.1152/ajpgi.00107.2007

[25] Welti J, Loges S, Dimmeler S, Carmeliet P. Recent molecular discoveries in angiogenesis and antiangiogenic therapies in cancer. The Journal of clinical investigation. 2013;123(8):3190–200.s 10.1172/JCI70212 23908119 PMC3726176

