# Supplementary figures and images for: Morphometric and Quantitative Immunohistochemical Analysis of Disease-Related Changes in the Upper (Suburothelial) Lamina Propria of the Human Bladder Dome
Source: PLoS One. 2015 May 14;10(5):e0127020. doi: 10.1371/journal.pone.0127020 (PMC4431865; doi:10.1371/journal.pone.0127020)

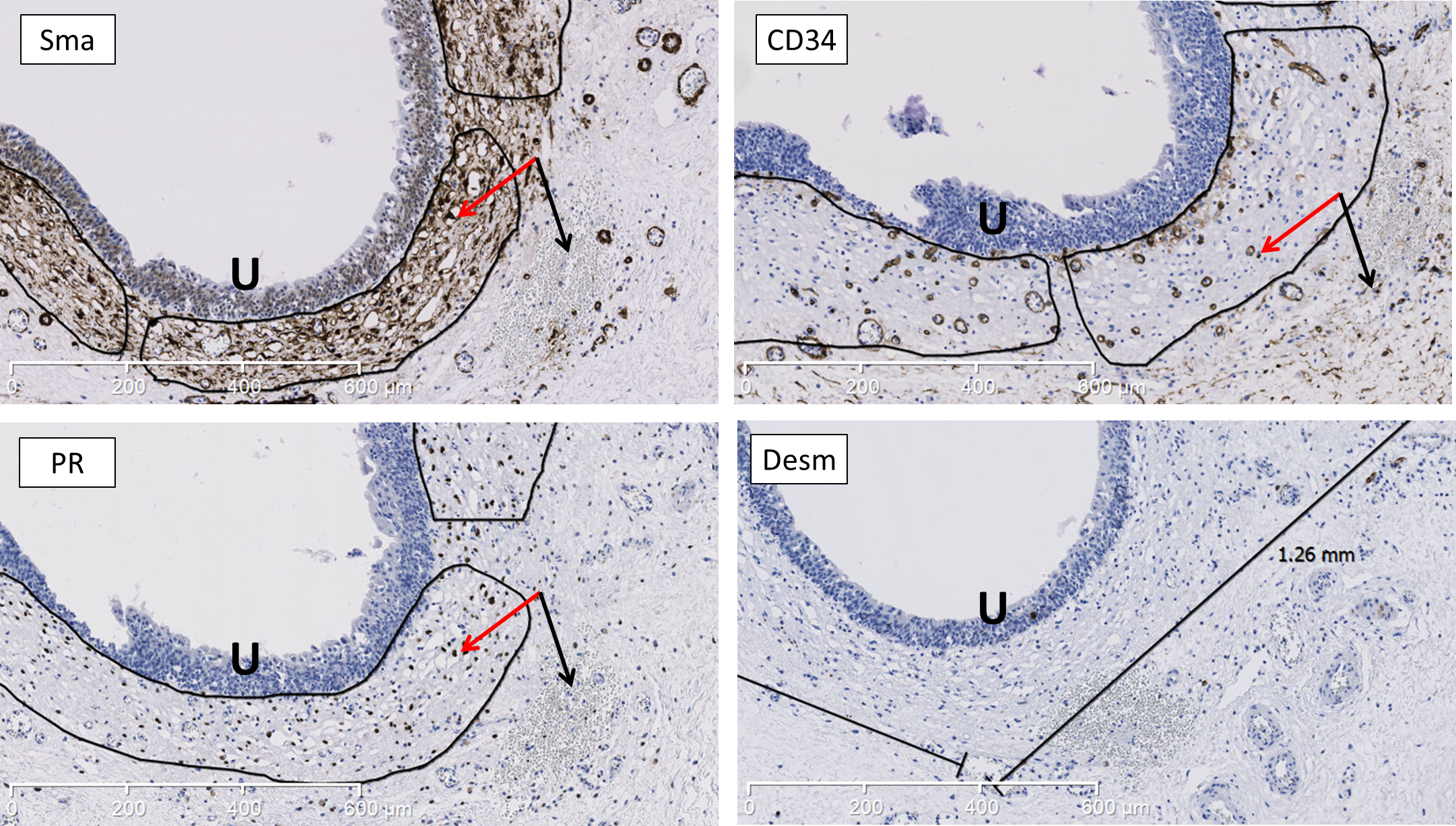

Supplement: S1 Fig — Illustrative figure that shows the annotation methodology for SMA+/CD34-/PR+ ULP IC, CD34+ blood vessels and desmin+ MM in the ULP area (all illustrations come from the same sample area). ULP IC areas are encircled by black diagrams. For SMA, PR and CD34 the same ULP IC areas were annotated on images of serial tissue slides, while for desmin the underlying presence or absence of MM was annotated with free hand diagrams and rulers respectively. The area on the present tissue slide shows absence of MM, resulting in annotation with rulers only. Black and red arrows indicate the differences between ULP IC and DLP IC respectively. All annotations were extracted in a second phase for morphometry and quantitative staining analysis. U is urothelium. Scale bars indicate 600μm. (TIF) [file pone.0127020.s001.tif]

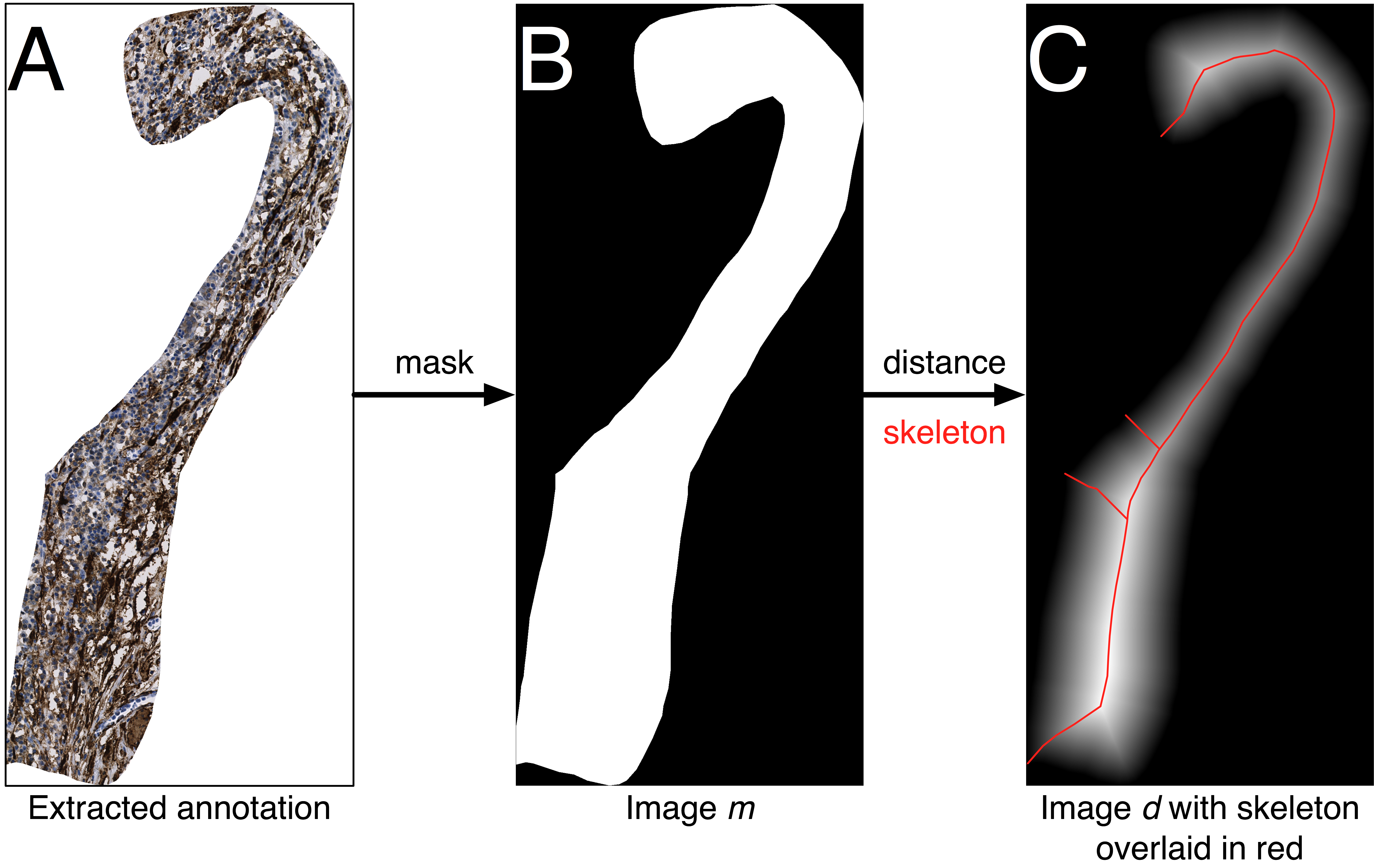

Supplement: S2 Fig — (A) ULP IC annotation extracted as a bitmap image. (B) Binary mask (m) identifying ULP IC area (in white on a black background). (C) Distance transform applied on image m to produce image d and its skeleton as the ridges of the distance function (overlaid in red). (TIFF) [file pone.0127020.s002.tiff]
