# Supplementary material for: Morphometric and Quantitative Immunohistochemical Analysis of Disease-Related Changes in the Upper (Suburothelial) Lamina Propria of the Human Bladder Dome
Source: PLoS One. 2015 May 14;10(5):e0127020. doi: 10.1371/journal.pone.0127020 (PMC4431865; doi:10.1371/journal.pone.0127020)
Supplement: S1 File — (DOCX) [file pone.0127020.s003.docx]

**Supplementary data**

*- Calculation of MTH and MA:*

Our extraction tool generates two images per annotation. The first image shows the annotated tissue on a white background (see supplementary figure 2A). The second image, which we named *m* (see Figure S1B), is a binary image of the same size than the first showing the annotated tissue in white on a black background. A distance transform is then applied to the second image, producing image *d* (see supplementary figure 2C), and the skeleton, *S*, is extracted (overlaid in red in supplementary figure 2C). Two thickness measures were then computed in Python using SciPy^1^ and the scikit-image image processing toolkit^2^.

The first measure, MA, corresponds to the height of a rectangle of same area and length than the annotation and is defined as:

$$MA=\frac{1}{N}\sum_{i\in m} m_{i}$$

where *m_i_* is the value of pixel *i* in image *m* and *N* is the number of pixels that belong to the extracted skeleton *S*, i.e. the skeleton length.

The second measure, MTH, corresponds to twice the average distance of the pixels that belong to the skeleton (i.e. included in the set *S*) to their nearest annotation border. Because the pixels in *S* are located about at the annotation center, a factor 2 is needed to obtain a better approximation of the thickness:

$$MTH=\frac{2}{N}\sum_{i\in S} d_{i}$$

where *d_i_* is the value of the distance of pixel *i* to the closest border of the annotation and *N* is the skeleton length.

*- Calculation of STD_TH:*

The STD_TH measure was extracted to evaluate thickness variations. STD_TH is the standard deviation of the annotation thickness measured along the skeleton:

$$STD\_TH=\frac{1}{N}\sqrt{\sum_{i\in S} \left( {2d}_{i}-MTH \right)^{2}}$$

All the thickness-related measures are expressed in pixels, where 1 pixel = 0.46µm (at 20X magnification)

1. Jones E, Oliphant T, Peterson P. SciPy: Open source scientific tools for Python. 2011. Available: http://www.scipy.org. Accessed 9 April 2014.

2. van der Walt S, Schönberger JL, Nunez-Iglesias J, *et al*. scikit-image: Image processing in Python. *PeerJ PrePrints* 2014; **2**:e336v2. Available: [http://dx.doi.org/10.7287/peerj.preprints.336v2. Accessed 9 April 2014](http://dx.doi.org/10.7287/peerj.preprints.336v2.%20Accessed%209%20April%202014).
